# Machine learning-based prediction of invasiveness in lung adenocarcinoma presenting as ground-glass nodules using radiomics and clinical CT features

**DOI:** 10.1186/s12885-025-14983-3

**Published:** 2025-11-03

**Authors:** Mingzhi Lin, Longqian Li, Yiming Hui, Bin Li, Yue Li, ChongRui Li, Zhizhong Zheng, Zhuowen Yang

**Affiliations:** 1 Department of Thoracic Surgery, The Second Hospital & Clinical Medical School, Lanzhou University, 82 Cuiyingmen, Chengguan District, Lanzhou, 730030 China; 2 The Affiliated Yantai Yuhuangding Hospital of Qingdao University, Yantai, Shandong China

**Keywords:** Lung adenocarcinoma, Ground-Glass nodule, Principal component analysis, Machine learning, Radiomics

## Abstract

**Background:**

Lung adenocarcinoma(LA), the predominant histological subtype of lung cancer, frequently manifests as ground-glass nodules (GGNs) on computed tomography. Preoperative discrimination of invasiveness—critical for guiding surgical and therapeutic decisions—remains challenging due to subjective radiological assessment and limited sensitivity of conventional methods. This multicenter study aimed to develop a robust, non-invasive predictive framework integrating radiomics and clinical CT features using machine learning (ML) to stratify GGN-associated LA invasiveness.

**Methods:**

A retrospective dual-cohort analysis was conducted on 357 patients with pathologically confirmed LA. The primary cohort (*n* = 312) was randomly divided into a training cohort (*n* = 249) and a test cohort (*n* = 63) at an 8:2 ratio. The external validation cohort consisted of 45 patients. Radiomics features (*n* = 1129) were extracted from High Resolution CT (HRCT), and clinical CT features (*n* = 16) were evaluated by blinded radiologists. Principal component analysis (PCA) and least absolute shrinkage and selection operator (LASSO) were respectively used for dimensionality reduction of radiomics features and five ML algorithms (XGBoost, SVM, Random Forest, Logistic Regression, LightGBM) were trained to predict invasiveness (low: minimally invasive adenocarcinoma/Grade 1 invasive adenocarcinoma; high: Grade 2/3 invasive adenocarcinoma). Model performance was assessed using Area Under the Curve (AUC), sensitivity, specificity, and Decision Curve Analysis. The calibration curve was plotted, and SHapley Additive exPlanations methods were used to interpret the predictive models.

**Results:**

The Random Forest model In the Clinical CT Features-PCA radiomics model performed the best, with an AUC value of 0.854 for the training cohort, 0.769 for the test cohort, and 0.778 for the external validation cohort. Key predictive features included PCA-derived radiomic components and clinical CT Features. Clinical CT Features-PCA Radiomics RF model significantly outperformed clinical-only models and Clinical CT Features-LASSO Radiomics Model, showing superior predictive ability.

**Conclusions:**

Integration of radiomics and clinical CT features via ML, particularly RF, enables accurate preoperative prediction of LA invasiveness in GGNs. This approach enhances objectivity over conventional radiological assessment and may optimize personalized treatment strategies. Further validation in larger, prospective cohorts is warranted to confirm clinical utility.

**Supplementary Information:**

The online version contains supplementary material available at 10.1186/s12885-025-14983-3.

## Introduction

Lung cancer remains the most prevalent cancer globally and is the leading cause of cancer-related mortality [1]. Early-stage lung cancer typically presents as pulmonary nodules, which are categorized into solid nodules (SN) and ground-glass nodules (GGN) based on their composition and radiological characteristics. Ground-glass nodules are further subdivided into pure ground-glass nodules (pGGN) and part-solid nodules (PSN), with malignant part-solid nodules often manifesting as lung adenocarcinoma (LA) [2–4]. Lung adenocarcinoma is the most common histological subtype of lung cancer, accounting for approximately 55-60% of all cases [5]. Due to underlying medical conditions and limited patient awareness, many individuals are diagnosed with advanced lung cancer at their first medical visit [6]. Treatment options for advanced lung cancer are highly restricted, with a five-year cumulative survival rate of only 19% in China [7]. However, early detection through screening can substantially improve the prognosis and survival rates of patients. Consequently, early screening and diagnosis of lung cancer play a pivotal role in reducing mortality and enhancing survival outcomes. Current common early screening methods include low-dose spiral CT (LDCT) scanning, biomarker testing, and tumor-associated antibody screening, among others. LDCT has become increasingly widespread in lung cancer screening and is now the recommended method in China [7, 8]. Despite its broad application, LDCT faces several challenges, such as the difficulty in distinguishing indeterminate pulmonary nodules and the risk of overdiagnosing indolent tumors. The evaluation of malignancy and invasiveness of ground-glass nodules frequently depends on the subjective judgment of radiologists, considering factors such as nodule morphology, size, proportion of solid components, and surrounding characteristics [9]. However, the morphological features distinguishing glandular precursor lesions, minimally invasive adenocarcinoma (MIA), and invasive adenocarcinoma (IA) remain unclear [10]. Some early-stage IA may present as pGGN or exhibit a low proportion of SN, which can easily lead to diagnostic errors [11]. At the same time, biomarker and tumor antibody screenings suffer from issues of insufficient sensitivity and specificity [12]. Additionally, the infiltration status of GGN shows significant variability [13], leading to limitations in existing methods for evaluating GGN infiltration, particularly in the preoperative setting.

Radiomics, as an emerging technology, extracts a large number of quantitative features from medical images, revealing potential information that traditional imaging examinations may not detect. These features may include parameters such as shape, texture, edges, and density, and radiomics has shown great potential in lung cancer screening and diagnosis [14, 15]. Through radiomics analysis, we can not only quantitatively assess the nature of nodules but also provide important information about tumor biological behavior, offering a more precise auxiliary tool for clinical decision-making. Therefore, this study aims to address these limitations by proposing a new framework that combines CT-based radiomics with deep learning features, focusing on lung adenocarcinoma presenting as ground-glass nodules GGN, and conducting a retrospective, dual-center clinical study. By integrating radiomics analysis with clinical parameters for an in-depth study of CT scan images, we plan to predict the invasiveness of lung adenocarcinoma preoperatively and non-invasively, assisting surgeons in making more precise and personalized surgical and treatment decisions for different patients.

## Materials and methods

### Patient selection

This study was conducted as a multicenter retrospective analysis, in accordance with relevant laws and regulations, and was approved by the ethics committee. The ethics committee unanimously agreed to waive the informed consent requirement and approved the study protocol (Second Hospital of Lanzhou University, approval number: 2024A-919; Yantai Yuhuangding Hospital, approval number: 2025-589). All data were anonymized and did not contain any personal information of the patients, effectively preventing reverse identification. Data access is strictly controlled, with access granted only to authorized researchers.

Clinical data from 312 patients who underwent GGN surgery at the Second Hospital of Lanzhou University from January 2020 to February 2025 were collected as the primary cohort. Additionally, data from 45 patients who received the same treatment at Yantai Yuhuangding Hospital during the same period were collected as the validation cohort.

Inclusion criteria included: (1) patients who underwent lung cancer surgery with complete surgical records of pulmonary nodule resection and a postoperative pathological report confirming lung adenocarcinoma; (2) CT examination within 1 week before surgery, with image slice thickness ≤1.25mm and a diagnosis of GGN; (3) no prior antitumor therapy before surgery; and (4) GGN diameter less than 3 cm. Exclusion criteria included: (1) poor image quality or significant artifacts; (2) a history of malignant tumors before surgery or prior treatment with other therapies; and (3) nodules identified as metastases from tumors in other locations.

### CT scanning protocol

CT imaging was conducted using various systems: the Discovery CT750 HD (GE Healthcare), Philips iCT 256 (Koninklijke Philips N.V.), and Somatom Sensation 64 (Siemens, Erlangen, Germany). The scan parameters were as follows: the tube voltage was set at 120 kVp, with the tube current ranging from 150 to 200 mA. Both the axial image layer thickness and spacing were maintained at 5 mm, while the reconstruction layer thickness and interval were both set to 1.25 mm.

### Clinical and CT image features analysis

The imaging semantic features of pulmonary nodules were analyzed by two mid-level radiologists and thoracic surgeons and reviewed by a senior radiologist with 15–30 years of experience. The radiologists were blinded to the clinical findings and histological results. Disagreements regarding the imaging features were resolved through discussion. The images were viewed at the lung (window width, 1500 HU; level,-600 HU) and mediastinal (window width, 350 HU; level, 50 HU) windows. The CT features recorded in the analysis were as follows: (1) nodule location (left and right lungs, upper, middle, and lower lobes), including whether located in the upper lobe; (2) nodule maximal long-axis diameter; (3) solid component maximal diameter; (4) other characteristics (lobulation, spiculation, vacuole, pleural indentation, air bronchogram, and vascular convergence). Clinical data of patients, including sex and age, were collected and recorded.

### Image preprocessing

Before segmentation and feature extraction, image preprocessing is essential to reduce data heterogeneity across different CT devices. First, voxel size was resampled to 1× 1 × 1 mm³, and anisotropic images were homogenized to minimize voxel size dependence of radiomics features. The voxel intensity values were discretized with a fixed bin width of 25 HU to reduce noise and standardize intensity for stable resolution. Signal intensity was normalized to 1-500 HU to minimize differences from various machines. Finally, Z-score normalization was applied to standardize gray values, reducing the impact of parameter inconsistencies on feature variation [16].

### Pulmonary Nodule Segmentation and Feature Extraction

**Fig. 1:**
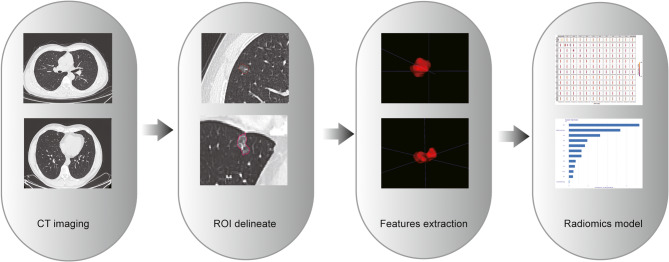
Radiomics analysis process. Abbreviations: ROI, region of interest

CT images of pulmonary nodules were segmented using ITK-SNAP version 4.0. Thin-slice CT images were imported into ITK-SNAP, with the window width set to 1500 HU and the window level set to -600 HU for optimal image visualization. The regions of interest (ROI) for all lesions were manually delineated through consensus by a mid-level radiologist and a thoracic surgeon, and were subsequently reviewed and confirmed by a senior radiologist with 15 years of experience after undergoing standardized training prior to segmentation. The final ROIs represent a consensus from all three parties, and therefore, independent consistency testing was not performed. Radiomics features were extracted from the GGN regions using the "PyRadiomics" package in Python 3.11.0. yielding a total of 1129 features, including first-order features, histogram features, texture features, and density features. For example, features extracted from the Gray Level Co-occurrence Matrix (GLCM), Gray Level Run Length Matrix (GLRLM), and Gray Level Size Zone Matrix (GLSZM) were included in the analysis. (The process of extracting radiomics features is shown in Fig. 1).

### Feature selection and model construction

Two cohorts from different centers were utilized: a primary cohort and a validation cohort. Initially, important variables were independently selected from both cohorts. Subsequently, the false discovery rate (FDR) correction was applied within the primary cohort to control for multiple testing. The significant variables identified from both cohorts were then combined to form the union of the significant features. To further refine the selected features, Pearson correlation analysis was conducted to filter out highly correlated radiomics features. This process ensured that only the most relevant and non-redundant features were retained for subsequent analysis. For the clinical CT features, the Variance Inflation Factor (VIF) was calculated, and clinical variables with a VIF less than 5 were retained to reduce the impact of multicollinearity on the model. For radiomic features, to construct a machine learning prediction model with strong generalizability and to simplify the feature space while enhancing computational efficiency, we performed dimensionality reduction on the radiomics features using both Least Absolute Shrinkage and Selection Operator(LASSO)regression and principal component analysis (PCA) [17] to build machine learning prediction models with strong generalizability and improved computational efficiency. LASSO regression, by incorporating L1 regularization, effectively selects relevant features, automatically removing irrelevant ones, reducing model complexity, and preventing overfitting. PCA, on the other hand, maps the original features to a new orthogonal space through linear transformation, preserving the most significant information while maximizing the variance in the data [18, 19]. We compared the performance of these two dimensionality reduction methods in the model to determine which approach was more effective in enhancing predictive performance. The process for selecting clinical features mirrored that of the radiomics features. Five machine learning algorithms used in this study (XGBoost, support vector machine (SVM), random forest (RF), logistic regression (LR), and Light Gradient Boosting Machine (LightGBM) to construct the final model. T The primary cohort data were divided into training and test sets in an 8:2 ratio, with the training cohort containing 249 samples and the test cohort containing 63 samples. For the training set, five-fold cross-validation and grid search were applied to identify the optimal hyperparameters. Sensitivity and specificity were evaluated using the Receiver Operating Characteristic (ROC) curve. External validation was conducted using the validation cohort to assess the model's discriminative power and clinical utility. The most optimal features and the best model were selected, and ROC curves, calibration curves, and Decision Curve Analysis (DCA) curves were generated and visualized through SHapley Additive exPlanations (SHAP) to further elucidate the contribution of each feature to the model's predictions.

### Statistical analysis

Statistical analyses were conducted using SPSS (version 26.0) and R (version 4.2.0) software. For continuous data, the Shapiro-Wilk test for normality were first performed. For data that followed a normal distribution with equal variances, independent t-tests were performed, with results presented as mean ± standard deviation. Categorical data were presented as frequencies and percentages, with group differences assessed using the Chi-square test or Fisher's exact test. The diagnostic performance of the model was evaluated using metrics such as the AUC, accuracy, sensitivity, and specificity. SHAP visualization was employed to interpret the contribution of each feature to the model's predictions.

## Results

### General information and Clinical CT Features

Table 1 presents an overview of the clinical characteristics of each patient. A total of 357 patients participated in this study, with 312 from the primary cohort and 45 from the validation cohort. In the primary cohort, the ages of the low-invasive group and high-invasive group were 55.1 ± 9.79 years and 58.21 ± 9.4 years, respectively, with a statistically significant difference (*P* = 0.01). In the validation cohort, the ages of the low-invasive group and high-invasive group were 58.1 ± 11.45 years and 62.56 ± 7.72 years, respectively, with no statistically significant difference (*P* = 0.171). Regarding gender distribution, in the primary cohort, 66.9% (113/169) of the low-invasive group were female, and 62.9% (90/143) of the high-invasive group were female, with no significant difference (*P* = 0.469). In the validation cohort, 65.5% (19/29) of the low-invasive group were female, and 62.5% (10/16) of the high-invasive group were female, with no significant difference (*P* = 0.842).

**Table 1:** Baseline characteristics and clinical CT features of patients in the primary cohort and validation cohort

	Primary cohort(*n*=312)				Validation cohort(*n*=45)		
EVENT	Low-Invasive Group	High-Invasive Group	*P*-value	FDR	Low-Invasive Group	High-Invasive Group	*P*-value
Age	55.1 ± 9.79	58.21 ± 9.4	0.010	0.014	58.1 ± 11.45	62.56 ± 7.72	0.171
Sex			0.469	0.58			0.842
Female	113 (66.9%)	90 (62.9%)			19 (65.5%)	10 (62.5%)	
Male	56 (33.1%)	53 (37.1%)			10 (34.5%)	6 (37.5%)	
Lesion Location			0.552	0.586			0.682
Right Upper Lobe	70 (41.4%)	49 (34.3%)			10 (34.5%)	6 (37.5%)	
Right Middle Lobe	9 (5.3%)	13 (9.1%)			1 (3.4%)	0 (0.0%)	
Right Lower Lobe	24 (14.2%)	23 (16.1%)			2 (6.9%)	1 (6.2%)	
Left Upper Lobe	48 (28.4%)	40 (28.0%)			10 (34.5%)	3 (18.8%)	
Left Lower Lobe	18 (10.7%)	18 (12.6%)			6 (20.7%)	6 (37.5%)	
Location in Upper Lobe			0.096	0.153			0.539
No	51 (30.2%)	56 (39.2%)			10 (34.5%)	7 (43.8%)	
Yes	118 (69.8%)	87 (60.8%)			19 (65.5%)	9 (56.2%)	
Density			< 0.001	< 0.001			0.189
Pure Ground Glass	99 (58.6%)	46 (32.2%)			13 (44.8%)	4 (25.0%)	
Mixed Ground Glass Opacity	70 (41.4%)	97 (67.8%)			16 (55.2%)	12 (75.0%)	
Irregularity			< 0.001	< 0.001			0.186
Absence	124 (73.4%)	72 (50.3%)			15 (51.7%)	5 (31.2%)	
Presence	45 (26.6%)	71 (49.7%)			14 (48.3%)	11 (68.8%)	
Spiculation			< 0.001	< 0.001			0.225
Absence	136 (80.5%)	79 (55.2%)			26 (89.7%)	12 (75.0%)	
Presence	33 (19.5%)	64 (44.8%)			3 (10.3%)	4 (25.0%)	
Lobulation			< 0.001	0.002			0.082
Absence	129 (76.3%)	84 (58.7%)			15 (51.7%)	4 (25.0%)	
Presence	40 (23.7%)	59 (41.3%)			14 (48.3%)	12 (75.0%)	
Pleural indentation			< 0.001	0.001			0.463
Absence	111 (65.7%)	66 (46.2%)			16 (55.2%)	7 (43.8%)	
Presence	58 (34.3%)	77 (53.8%)			13 (44.8%)	9 (56.2%)	
Vacuum			0.42	0.553			0.017
Absence	144 (85.2%)	117 (81.8%)			28 (96.6%)	11 (68.8%)	
Presence	25 (14.8%)	26 (18.2%)			1 (3.4%)	5 (31.2%)	
Cavity			0.715	0.912			1
Absence	160 (94.7%)	134 (93.7%)			27 (93.1%)	15 (93.8%)	
Presence	9 (5.3%)	9 (6.3%)			2 (6.9%)	1 (6.2%)	
Air Bronchogram Sign			< 0.001	< 0.001			0.002
Absence	91 (53.8%)	31 (21.7%)			24 (82.8%)	6 (37.5%)	
Presence	78 (46.2%)	112 (78.3%)			5 (17.2%)	10 (62.5%)	
Vascular convergence Sign			0.012	0.026			0.005
Absence	89 (52.7%)	55 (38.5%)			18 (62.1%)	3 (18.8%)	
Presence	80 (47.3%)	88 (61.5%)			11 (37.9%)	13 (81.2%)	
Consolidation Tumor Ratio	0.2 ± 0.26	0.39 ± 0.32	< 0.001	< 0.001	0.3 ± 0.27	0.26 ± 0.25	0.491
Maximum Diameter	15.01 ± 6.5	17.5 ± 5.73	< 0.001	< 0.001	13.38 ± 4.47	15.56 ± 4.55	0.110
Maximum Diameter of Solid Component	3.3 ± 4.79	7.45 ± 7.08	< 0.001	< 0.001	4.31 ± 4.4	3.94 ± 4.33	0.780

Several clinical CT features displayed significant differences between the two groups. In the primary cohort, the low-invasive group exhibited significantly more frequent absence of abnormalities in irregularity, lobulation, pleural retraction, Air Bronchogram Sign, and Vascular Bundle sign (P < 0.001), whereas the high-invasive group demonstrated a higher frequency of vacuum (*P* = 0.42). In the validation cohort, tlow-invasive group and high-invasive group exhibited minor differences in most features, with only vacuum and Air Bronchogram Sign showing statistically significant differences (*P* = 0.017 and *P* = 0.002). These results suggest that there are distinct differences in imaging features between the two groups, although variability may exist across different datasets. The statistics for the radiomic features are provided in the supplementary table (Table S1).

### Features selection

**Fig. 2:**
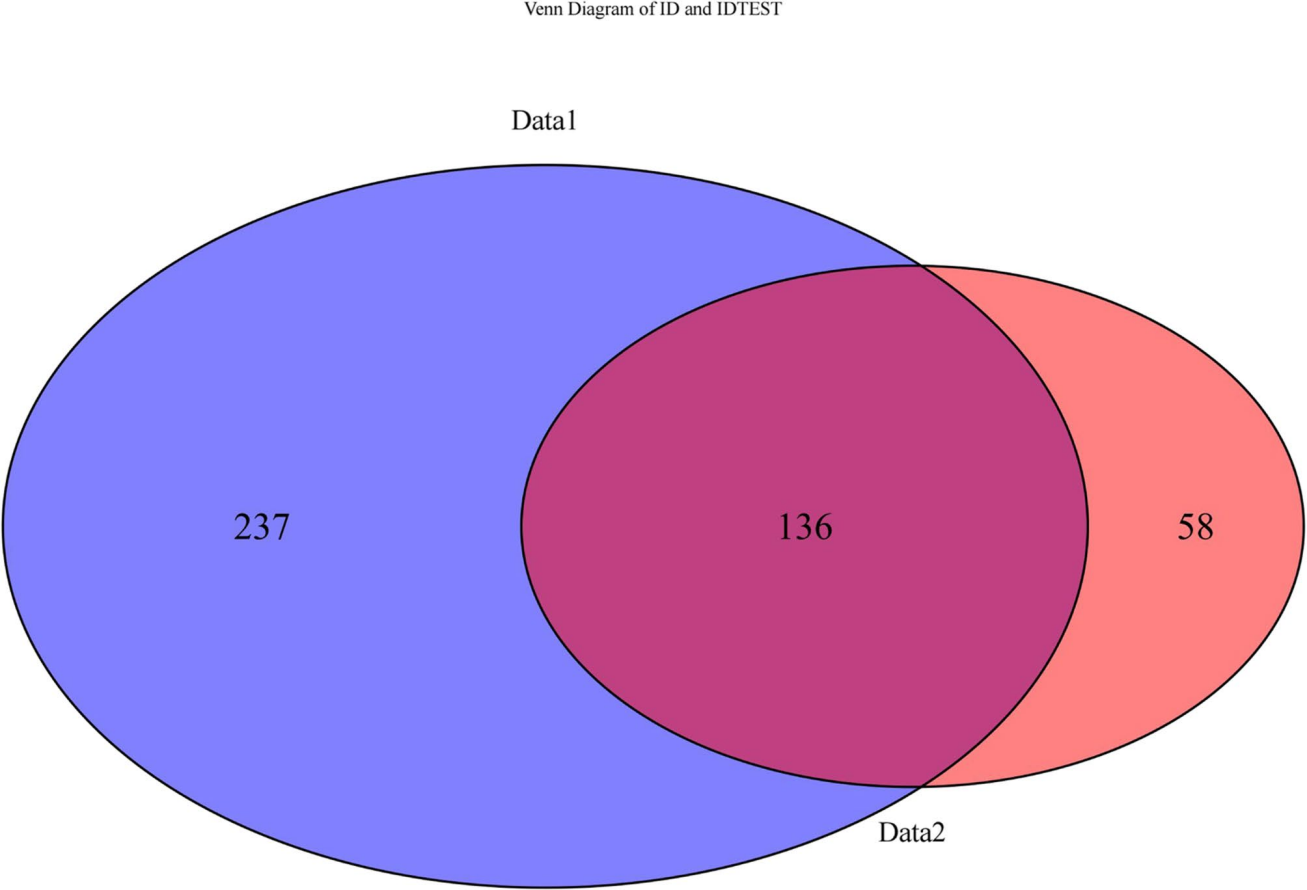
Venn diagram showing overlapping features between Primary cohort(left) and validation cohort(right)

**Fig. 3:**
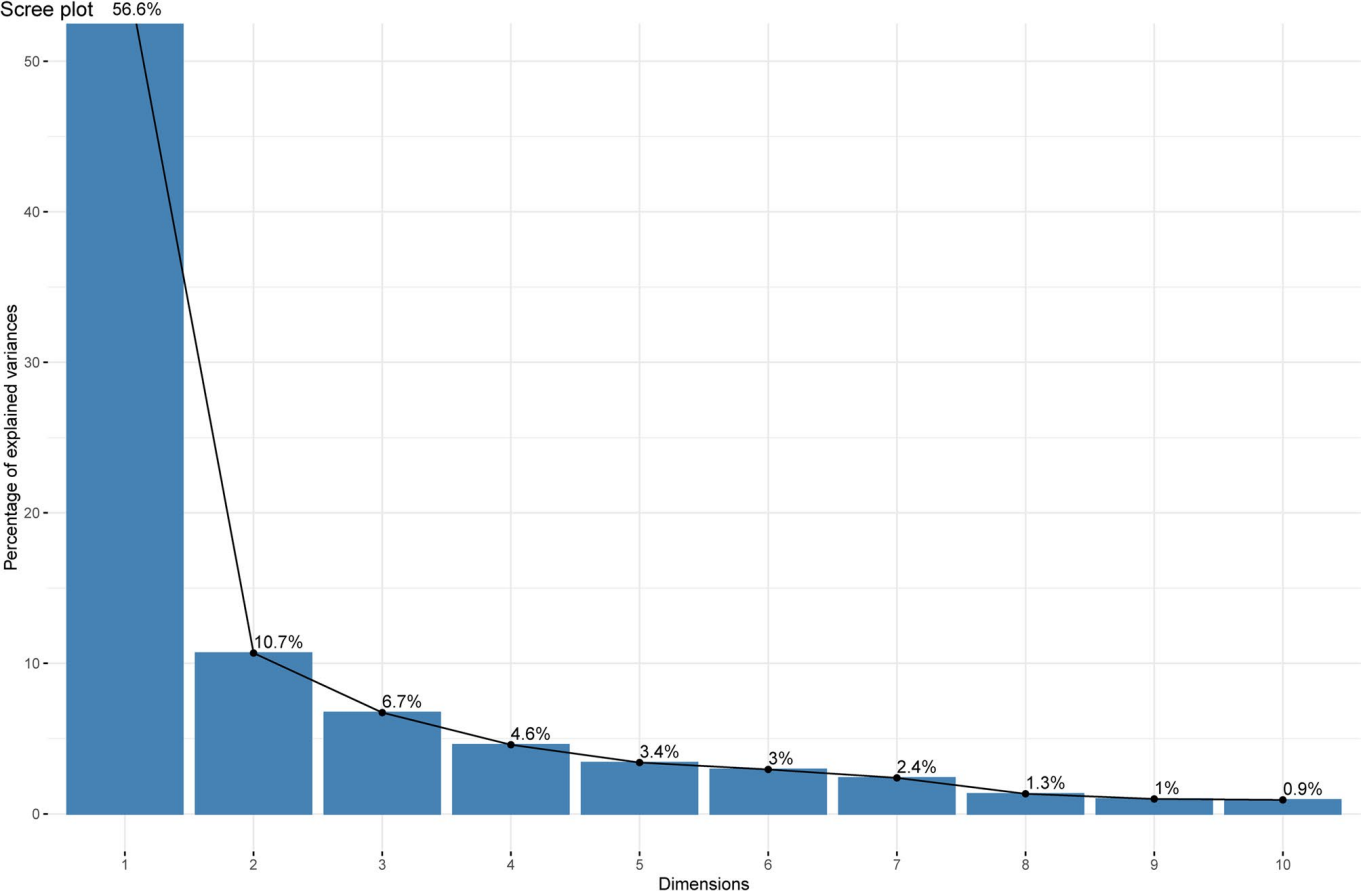
Scree plot representing the top ten contributing principal components

**Fig. 4:**
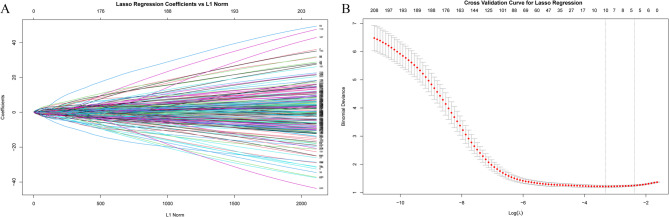
Screening of variables based on Lasso regression. A: Lasso Coefficient Path,B: Cross-validation curve for Lasso regression.λ.min= 0.03647

In this study, a total of 1129 high-throughput radiomic features and 16 clinical and CT features were extracted. To develop a machine learning prediction model with robust generalizability, statistical methods (such as t-tests and Chi-square tests) were first applied to identify 808 significant variables in the primary cohort. After performing the FDR correction, 774 variables with FDR < 0.05 were retained for further analysis. Subsequently, Pearson correlation analysis was performed, and variables with a correlation coefficient (r) greater than 0.2 were selected [20]. A heatmap of the top 20 most strongly correlated variables was generated, as shown supplementary materials (Figures S1 and S2). We included variables that were significantly associated with the outcome in each of the two cohorts and took their intersection, resulting in a total of 422 variables. Figure 2 shows the Venn diagram of the included variables. Subsequently, we incorporated 289 variables that were not significant in both cohorts into the analysis (Tables S4). To further simplify the feature space and enhance computational efficiency, we employed two feature selection methods: PCA and LASSO. First, we performed PCA on the radiomic features and selected the top 10 principal components with the highest contribution for subsequent analysis (PCA explained variance and loadings matrix in the supplementary table (Tables S2 and S3). As shown in Fig. 3, these 10 principal components explained 90.5% of the total variance. Second, we applied LASSO regression (α = 1) to further reduce the dimensionality of the radiomic features and employed five-fold cross-validation to optimize the model. During the cross-validation process, the optimal λ value (λ.min = 0.03647) was selected, resulting in the retention of 10 key radiomic features (Fig. 4). The LASSO coefficient path and cross-validation curve are shown in Fig. 4. Finally, we employed five ML algorithms to construct three different predictive models, based on selected clinical CT features, clinical CT features combined with radiomic features reduced by PCA, and clinical CT features combined with radiomic features reduced by LASSO. The main cohort data is divided into an 8:2 ratio into the training cohort and the internal validation cohort and five-fold cross-validation with grid search was applied to the training set to determine the optimal hyperparameters. Sensitivity and specificity were assessed using the Receiver Operating Characteristic (ROC) curve, and external validation was performed using the validation cohort to evaluate the model's discriminative ability and clinical utility.We have created a feature selection flowchart (Figure S3) to clearly illustrate the entire process from raw data preprocessing.

**Fig. 5:**
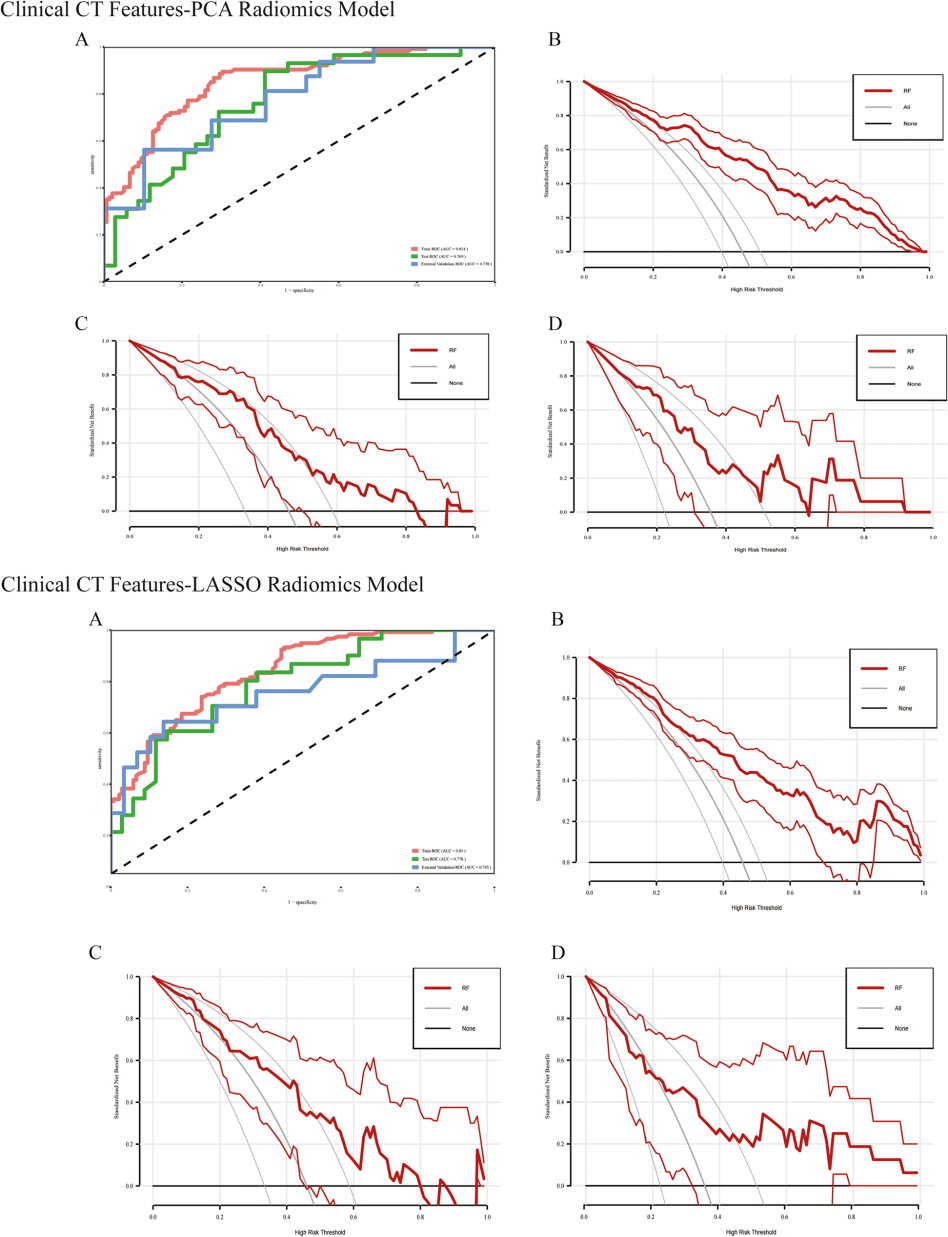
*R*OC curve and DCA of the RF mode*. ROC(Receiver Operating Characteristic) curve of the RF model, B.DCA (Decision Curve) Analysis of the RF model in the Primary cohort (Training cohort);* *C. DCA of the RF model in the Primary cohort (Test cohort); D. DCA of the RF model in the Validation cohort*

### Predictive model and validation test

Multiple features were integrated into various machine learning models. The conventional clinical CT feature model includes Consolidation Tumor Ratio (CTR), Maximum Diameter, Density (pGGN/PSN), Pleural Recession Sign, Vacuum, and Vascular Bundle Sign.LR demonstrated the best performance in the external validation cohort (AUC = 0.770), showcasing excellent generalization ability. Although its performance was moderate in the training (AUC = 0.702) and test cohort (AUC = 0.726), LR exhibited outstanding performance on new data, highlighting its strong predictive capability. We calculated the odds ratios (OR) and their 95% confidence intervals for each variable (Tables S5) to quantify the impact of each variable on the dependent variable and assess its statistical significance, thereby providing a scientific basis for interpreting and applying the model.In contrast, RF performed well in the training cohort (AUC = 0.771) but showed a decline in the external validation cohort (AUC = 0.654). Other models, such as SVM and LightGBM, also showed good performance (Table 2).

**Table 2:** Diagnostic performance of the clinical CT feature model distinguishing low invasiveness/high invasiveness

	Model	AUC	(95% CI)		Accuracy	Sensitivity	Specificity	PPV	npv
Training cohort (*n* = 249)	RF	0.771		0.712–0.828	0.711	0.675	0.741	0.688	0.73
	SVM	0.715		0.651–0.778	0.679	0.623	0.726	0.657	0.695
	LightGBM	0.780		0.724–0.836	0.711	0.693	0.726	0.681	0.737
	LR	0.702		0.637–0.766	0.671	0.500	0.815	0.695	0.659
	XGboost	0.714		0.649–0.778	0.691	0.518	0.837	0.728	0.673
Test cohort(*n* = 63)	RF	0.738		0.617–0.860	0.667	0.655	0.676	0.633	0.697
	SVM	0.708		0.582–0.835	0.635	0.621	0.647	0.600	0.667
	LightGBM	0.744		0.622–0.866	0.683	0.759	0.618	0.629	0.750
	LR	0.726		0.600-0.851	0.619	0.448	0.765	0.619	0.619
	XGboost	0.702		0.572–0.831	0.619	0.483	0.735	0.609	0.625
External validation cohort (*n*=45)	RF	0.654		0.484–0.824	0.622	0.500	0.690	0.471	0.714
	SVM	0.734		0.565–0.903	0.689	0.688	0.69	0.550	0.800
	LightGBM	0.639		0.457–0.821	0.578	0.500	0.621	0.421	0.692
	LR	0.770		0.629–0.912	0.733	0.438	0.897	0.700	0.743
	XGboost	0.690		0.533–0.847	0.644	0.313	0.828	0.500	0.686

*Abbreviations*: *AUC *Area under the curve, *PPV *Positive predictive value, *NPV *Negative predictive value, *CI *Confidence interval, *RF *Random forest, *SVM *Support vector machine, *LR *Logistic regression, *XGBoost *Extreme Gradient Boosting, *LightGBM *Light Gradient Boosting Machine

In models constructed using combined features multiple models demonstrated their respective advantages across different datasets (Table 3). In the predictive model combining CT features and PCA radiomics features, RF performed the best overall. It achieved an AUC of 0.854 (95% CI:0.808-0.901) in the training cohort and 0.778 in the external validation cohort, demonstrating excellent performance. RF exhibited a sensitivity of 0.895 in the training cohort, indicating a strong ability to correctly identify positive cases. Despite a decline in AUC during external validation, RF remained robust across multiple metrics, making it the most reliable model. LightGBM also showed good performance, with an AUC of 0.774 in the training cohort and 0.733 in the external validation cohort. While it demonstrated decent sensitivity and specificity, XGBoost performed well in the training cohort (AUC = 0.731), but its sensitivity and AUC declined in both the internal and external validation cohorts. LR showed moderate performance across all cohorts, with an AUC of 0.758 in the training cohort, but it did not surpass RF or GBM in performance. In the predictive model combining CT features with LASSO dimensionality reduction of radiomics features, RF still demonstrated the best performance. In the training cohort, RF achieved an AUC of 0.831 (95% CI: 0.781-0.879), with a sensitivity of 0.728 and specificity of 0.763, making it a reliable classification model. It also performed well in the test cohort (AUC = 0.778) and external validation cohort (AUC = 0.745), indicating good generalization capability. XGBoost showed good performance in the training cohort with an AUC of 0.752 and in the test cohort with an AUC of 0.745. However, it did not surpass RF in terms of AUC or overall consistency. LR had an AUC of 0.776 in the training cohort and 0.746 in the external validation cohort, but its sensitivity (0.754) and specificity (0.711) were lower than those of RF.

**Table 3:** Diagnostic performance of the combined features model distinguishing low invasiveness/high invasiveness

	Cohort	model	AUC	(95% CI)	Accuracy	Sensitivity	Specificity	PPV	NPV
Clinical CT Features-PCA Radiomics Model:	Training cohort	LR	0.758	0.699–0.818	0.719	0.614	0.807	0.729	0.712
	(*n* = 249)	XGBoost	0.731	0.668–0.795	0.711	0.693	0.726	0.681	0.737
		RF	0.854	0.808–0.901	0.787	0.895	0.696	0.713	0.887
		SVM	0.735	0.673–0.798	0.707	0.588	0.807	0.720	0.699
		LightGBM	0.774	0.716–0.831	0.707	0.746	0.674	0.659	0.758
	Test cohort	LR	0.718	0.589–0.847	0.683	0.621	0.735	0.667	0.694
	(*n* = 63)	XGBoost	0.695	0.563–0.827	0.635	0.655	0.618	0.594	0.677
		RF	0.769	0.685–0.853	0.714	0.931	0.529	0.628	0.900
		SVM	0.697	0.565–0.828	0.571	0.414	0.706	0.545	0.585
		LightGBM	0.757	0.638–0.876	0.714	0.759	0.676	0.667	0.767
	External validation cohort	LR	0.744	0.594–0.893	0.667	0.450	0.897	0.571	0.684
	(*n*=45)	XGBoost	0.720	0.569–0.871	0.622	0.625	0.621	0.476	0.750
		RF	0.778	0.638–0.918	0.644	0.75	0.586	0.600	0.810
		SVM	0.797	0.665–0.930	0.733	0.475	0.931	0.750	0.730
		LightGBM	0.733	0.571–0.895	0.644	0.688	0.621	0.500	0.783
Clinical CT Features-LASSO Radiomics Model	Training cohort	LR	0.776	0.718–0.833	0.731	0.754	0.711	0.688	0.774
	(*n* = 249)	XGBoost	0.752	0.691–0.813	0.731	0.667	0.785	0.724	0.736
		RF	0.831	0.781–0.879	0.747	0.728	0.763	0.722	0.769
		SVM	0.752	0.692–0.812	0.695	0.798	0.607	0.632	0.781
		LightGBM	0.781	0.725–0.838	0.719	0.711	0.726	0.686	0.748
	Test cohort	LR	0.762	0.641–0.882	0.714	0.724	0.706	0.677	0.750
	(*n* = 63)	XGBoost	0.745	0.625–0.866	0.667	0.655	0.676	0.633	0.697
		RF	0.778	0.665–0.892	0.714	0.793	0.647	0.657	0.786
		SVM	0.749	0.627–0.870	0.714	0.897	0.559	0.634	0.864
		LightGBM	0.774	0.660–0.889	0.683	0.828	0.559	0.615	0.792
	External validation cohort	LR	0.746	0.591-0.900	0.733	0.563	0.828	0.643	0.774
	(*n*=45)	XGBoost	0.777	0.623–0.931	0.733	0.75	0.724	0.600	0.840
		RF	0.745	0.574–0.916	0.667	0.688	0.655	0.524	0.792
		SVM	0.785	0.640–0.929	0.622	0.750	0.552	0.480	0.800
		LightGBM	0.719	0.547–0.891	0.756	0.688	0.793	0.647	0.821

*Abbreviations*: *AUC *Area under the curve, *PPV *Positive predictive value, *NPV *Negative predictive value, *CI *Confidence interval, *RF *Random forest, *SVM *Support vector machine, *LR *Logistic regression, *XGBoost *Extreme Gradient Boosting, *LightGBM *Light Gradient Boosting Machine, *PCA *Principal component analysis, *LASSO *Least absolute shrinkage and selection operator

Subsequently, we generated ROC curves, DCA curves, and calibration curves based on the RF model Fig. 5. The calibration curve demonstrates a high degree of consistency between predicted and observed values in both the Primary cohort and the Validation cohort (Figure S4). The results of the DCA indicate that the RF model provides better decision value across various high-risk thresholds compared to other approaches ("All" and "None"), especially in high-risk decision-making. Although the model's performance may exhibit some fluctuations at certain thresholds, it overall offers significant improvement in risk assessment. This suggests that the RF model can provide higher net benefits in real-world applications, helping decision-makers make more valuable decisions (Fig. 4).

### SHAP

In order to make the model and its prediction easier to understand, this study used the SHAP framework to quantify the contribution of features to the model prediction. CTR is the most important clinical CT feature, having the greatest impact on the model's performance, as confirmed by both methods. Other important features include plural indentation, and vscular convergence sign, which also contribute significantly to the prediction of Invasiveness in LA (Figure S5).

**Fig. 6:**
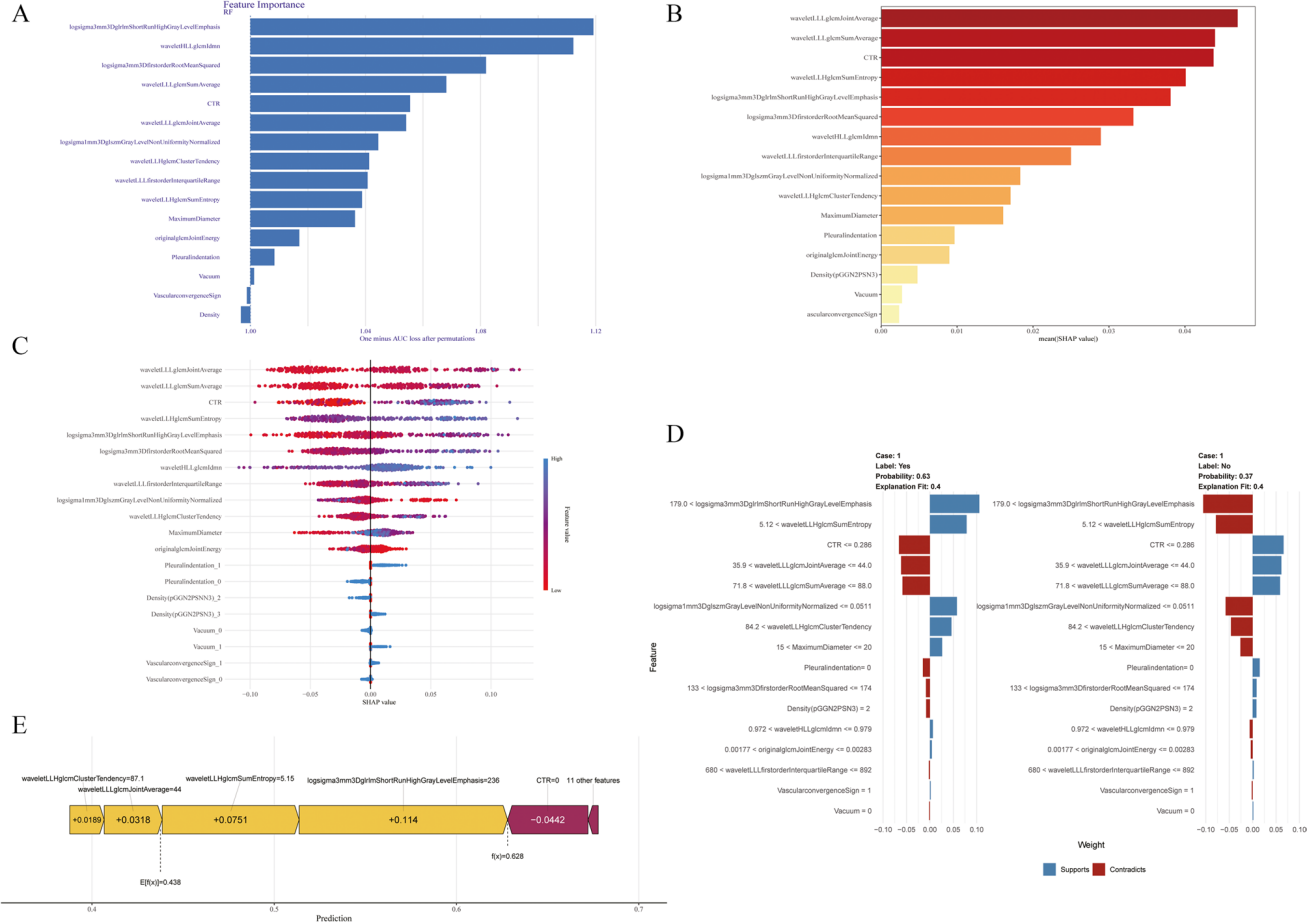
SHapley Additive exPlanations of Clinical CT Features-LASSO Radiomics Model (RF). Panel A: SHAP Feature Importance Plot.Panel B: SHAP Summary Plot.Panel C: SHAP Value Distribution Plot.Panel D: SHAP Decision Explanation Plot.Panel E: Local Interpretable Model-agnostic Explanations (LIME).0: Absence of Feature, 1: Presence of Feature

Figure 6 shows the five SHAP Panel of the best RF model in the Clinical CT Features-LASSO Radiomics Model. The feature importance plot in Fig. 6. Panel A ranks predictors by their influence on the model’s output. The bars for log-sigma-3-mm-3D GLRLM ShortRunHighGrayLevelEmphasis, wavelet-HLL GLCM Idmn, and CTR are the longest, indicating that these three variables exert the strongest average impact on the prediction. The SHAP mean value plot in Panel B displays the average SHAP value for each feature, the tallest bars—wavelet-LLL_GLCM_JointAverage, wavelet-LLL_GLCM_SumAverage, and CTR—indicate they exert the strongest overall influence on the model’s output, whereas the remaining predictors contribute progressively smaller effects. The SHAP value distribution plot in Panel C shows how much each feature influences the prediction and in which direction. The SHAP value on the x-axis measures the strength of this impact on the Invasiveness , while the y-axis ranks features by their value magnitude.Panel D: The SHAP decision weight plot illustrates the feature importance of a single sample based on SHAP values, where the blue and red bars represent the features that support and contradict the prediction, respectively. The left and right panels show the distribution of feature weights contributing to the model's prediction of the positive and negative classes, respectively. Local Interpretable Model-agnostic Explanations in Panel E shows each feature’s positive (yellow) or negative (purple) contribution to the model’s prediction is accumulated step by step, and the final prediction value is derived from the combined effect of all features.

**Fig. 7:**
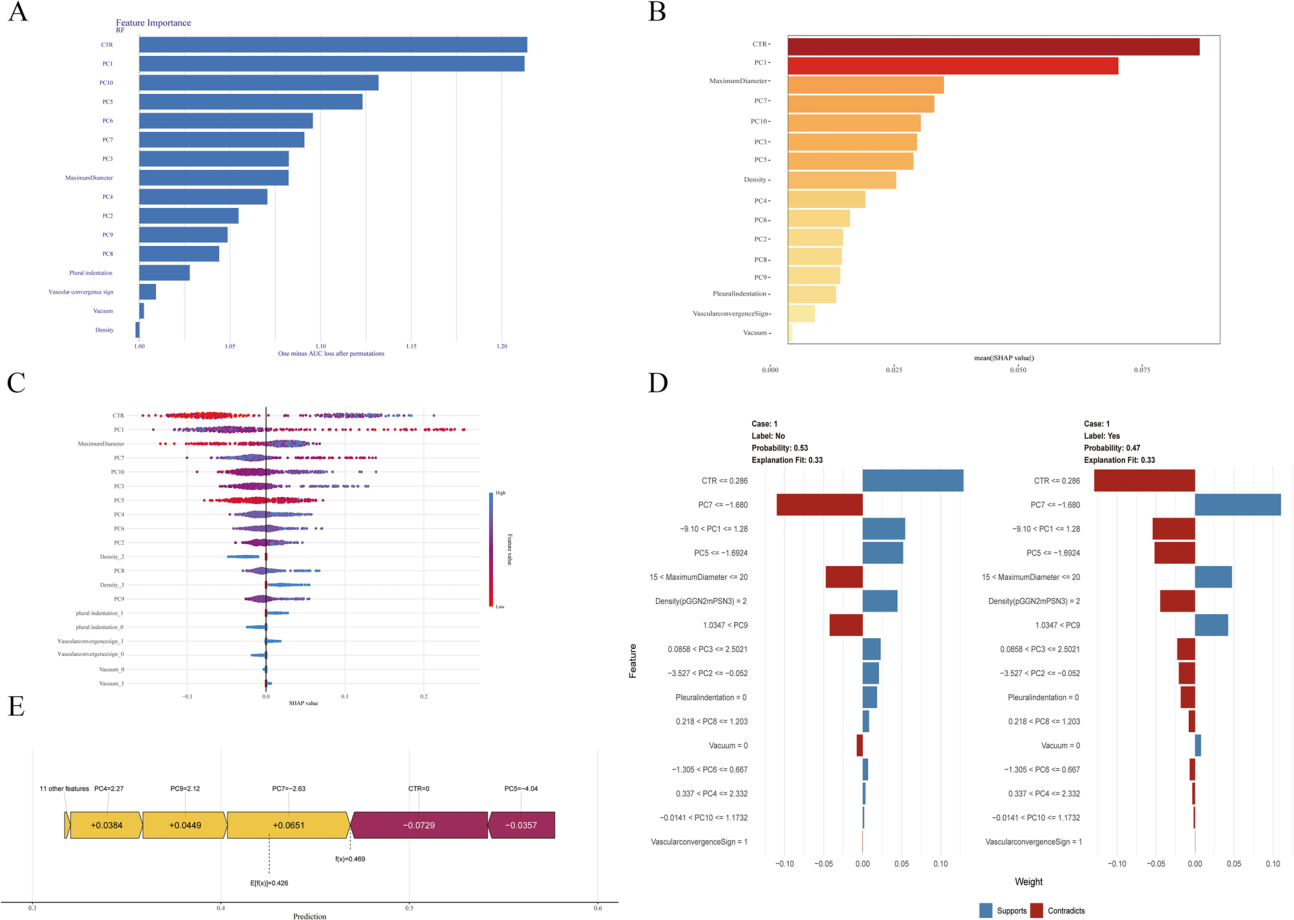
SHapley Additive exPlanations of Clinical CT Features-PCA Radiomics Model (RF)*. *Panel A: SHAP Feature Importance Plot.Panel B: SHAP Summary Plot.Panel C: SHAP Value Distribution Plot.Panel D: SHAP Decision Explanation Plot.Panel E: Local Interpretable Model-agnostic Explanations (LIME).0: Absence of Feature, 1: Presence of Feature

Figure 7 shows the five SHAP Panel of the best RF model in the Clinical CT Features-PCA Radiomics Model.The features with higher contributions are: CTR, PC1, and PC7. According to the PCA mapping, we can see that the top 10 features contributing to PC1 include Morphological Features, Texture Features, and Statistical Features, while the top 10 features contributing to PC7 are primarily Texture Features.

## Discussion

The 2021 World Health Organization (WHO) classification of LA delineates precursor glandular lesions (PGL), comprising atypical adenomatous hyperplasia (AAH) and adenocarcinoma in situ (AIS), with invasive subtypes encompassing MIA and IA [21]. IA is further subdivided into five histologic patterns: lepidic (LPA), acinar (APA), papillary (PAP), micropapillary (MPA), and solid predominant adenocarcinoma (SPA), each demonstrating distinct prognostic and therapeutic implications [22]. The International Association for the Study of Lung Cancer recently established a novel grading system for IA, stratifying tumors into three tiers: Grade 1: Lepidic-dominant tumors with <20% high-grade patterns；Grade 2: Acinar or papillary-dominant tumors with <20% high-grade patterns；Grade 3: Tumors exhibiting ≥20% high-grade patterns (solid, micropapillary, or complex glandular) [23]. Retrospective analyses [24–28] reveal comparable 5-year overall survival (OS) and recurrence-free survival (RFS) between MIA and Grade 1 IA, both characterized by favorable outcomes and low recurrence rates. These entities share clinicopathologic features of low-grade malignancy, limited invasiveness, and well-differentiated histology (primarily lepidic growth). Tumor cells in both subtypes demonstrate minimal cytologic atypia relative to normal tissue, reflecting an indolent biologic behavior. Consequently, neither necessitates aggressive therapeutic intervention, underscoring their association with reduced disease burden post-resection [28]. Grade 2 and Grade 3 IA typically exhibit higher malignancy and poorer prognosis. Tumor cells in Grade 2 are poorly differentiated, yet some degree of tissue structure is still discernible. In contrast, Grade 3 tumor cells typically lack distinct differentiation features, with considerable morphological deviation from normal cells, rapid growth, and greater invasiveness [27]. Treatment for such tumors generally requires a more comprehensive approach, including surgery, chemotherapy, targeted therapy, or immunotherapy. The prognosis is poor, with a higher risk of recurrence and metastasis. Therefore, combining Grade 2 and Grade 3 into a single group helps to reflect the shared characteristics of these tumors：greater invasiveness and more complex treatment requirements [29]. From a treatment perspective, Grade 1 and MIA patients typically only require local resection and are less Likely to need additional chemotherapy or radiotherapy. In contrast, Grade 2 and Grade 3 patients often require more aggressive treatment strategies [30, 31]. Therefore, classifying Grade 2 and Grade 3 tumors as high invasiveness and separating them from Grade 1 and MIA, while employing machine learning algorithms for non-invasive prediction of lung adenocarcinomas presenting as ground-glass nodules, will better reflect the distinct biological behavior, clinical treatment needs, and prognostic differences of these tumors. This classification not only aids clinicians in providing clear treatment directions but also helps in assessing patient prognosis.

In constructing the clinical feature model, we included features such as CTR, pleural indentation and vascular convergence sign. Multiple retrospective analyses [32–35] have shown that pleural indentation and vascular convergence sign are risk factors for predicting malignant pulmonary nodules, closely associated with the benign and malignant nature of pulmonary nodules. CTR is an important indicator for guiding physicians in the preoperative diagnosis and surgical decision-making for GGNs [36]. A higher CTR, indicating a larger solid component relative to the ground-glass area, is generally associated with an increased likelihood of malignancy. The JCOG0804 study [37] suggests that pulmonary nodules with CTR ≤ 0.25 and a total diameter ≤ 2 cm can be treated with wedge resection. The JCOG0802 [38] study goes further, aiming to evaluate whether segmentectomy can replace standard lobectomy for peripheral small lung cancers with CTR >0.5 and a diameter ≤ 2 cm. Our study also demonstrates that these features can be used to assess the invasiveness of malignant pulmonary nodules. However, the effectiveness of the clinical model built based on these features is not very high. Therefore, we have focused on exploring their potential in combination with radiomics features to develop a more precise model.

Radiomics can extract quantitative features from medical images at high throughput and, through objective and quantitative methods, reflect the tumor tissue heterogeneity that is invisible to the human eye [39, 40]. This aids in the development of predictive models and may improve diagnostic accuracy. These studies have yielded promising results in distinguishing between IAC and pre-invasive lesions, as well as in differentiating MIA from IAC. Zhang et al. [41] explored radiomics analysis of high-resolution CT imaging to predict the invasiveness of pure ground-glass nodules (pGGNs) in lung adenocarcinoma, analyzing data from 65 patients with a LASSO regression model. Specific radiomic features, such as"GLCMEntropy_angle135_offset1" and "Sphericity," effectively distinguished pre-invasive from invasive pGGNs, achieving an AUC of 0.824 in the testing group. This demonstrates the potential of radiomics as a non-invasive tool for preoperative assessment in lung adenocarcinoma. Lv et al. [42] used conventional CT, radiomics features, a combined CT and radiomics model, and a Delta-radiomics model to differentiate invasive from non-invasive lesions. The radiomics and combined models showed the highest diagnostic efficiency, with AUCs of 0.89 and 0.88 in the training and validation sets, respectively. The Delta-radiomics model achieved AUCs of 0.83 and 0.76, while the conventional model had lower AUCs of 0.78 and 0.76. The combined model outperformed the conventional one in distinguishing invasive pulmonary adenocarcinoma. According to the SHAP results, the radiomic features that contribute most to predicting invasiveness are predominantly GLRLM- and GLCM-based texture features. A previous study [43] demonstrated that CLCM feature assesses the spatial correlation between pixel gray levels in an image, helping to differentiate between different tissue types (such as tumor regions and normal tissue regions) as well as tumor heterogeneity. The differences in these features can reflect the histological grading of oral squamous cell carcinoma, and when combined with other radiomic features, they can enhance the accuracy of predicting tumor aggressiveness. Some researchers have also found that texture features contributing to the understanding of tumor heterogeneity and assisting in the characterization of malignant tissue ,especially when used in combination with other features, it can better assist in diagnosis malignancy of pulmonary nodules [44].

As scholars delve deeper into radiomics, increasing evidence suggests that PEF/CT radiomics may be used to investigate the molecular biological characteristics of tumors, such as PD-1/PD-L1 expression and tumor mutation burden, thereby providing a new approach for assessing the effectiveness of immunotherapy and predicting patient prognosis [45, 46]. Geng et al [47]. proposed a novel method combining multi-channel deep learning and Transformer Encoder, which can accurately diagnose whether non-small cell lung cancer patients undergoing neoadjuvant immunochemotherapy will achieve major pathological response. The multi-region radiomics model developed by Fan et al. [48] shows promising potential in predicting pathological complete response after neoadjuvant chemoradiotherapy for NSCLC, demonstrating excellent predictive performance across different specific subgroups. These studies indicate that radiomics can assist clinicians in accurately selecting appropriate candidates for neoadjuvant chemoradiotherapy and contribute to the advancement of precision medicine [49, 50].

In this study, we employed both PCA and LASSO in combination with various correction methods for radiomics feature dimensionality reduction, and integrated them with multiple machine learning models for analysis. Compared to LASSO, the PCA-based fusion feature model generally achieved slightly higher AUC values in both the training and validation sets, especially in the RF model. The PCA method provided more stable performance, while LASSO, although still effective, showed slight performance degradation in certain cases. LASSO may overlook some low-correlation but potentially important features during feature selection. Overall, both PCA and LASSO dimensionality reduction methods provided valuable features for the model, but the PCA-based model, particularly the RF model, demonstrated higher AUC values and more consistent results. Therefore, PCA shows a slight advantage over LASSO in terms of model accuracy and robustness. PCA [51] effectively retained the most representative radiomics features, reduced redundant information, and minimized the impact of noise on the model, thus improving both the efficiency and stability of processing complex imaging data. RF, as an ensemble learning method, enhances prediction accuracy and robustness by constructing multiple decision trees and aggregating their results [52, 53]. RF's built-in feature selection mechanism automatically identifies the most predictive features, thereby preventing overfitting and enhancing the model's generalization ability across different datasets. On the primary cohort’s training set, test set, and external validation set in this study, the RF model demonstrated excellent predictive performance and good stability, further validating the potential of integrating radiomic features with clinical CT features [54]. The integration of PCA-reduced radiomic features with clinical data not only enhanced the model's predictive capability but also helped reduce computational complexity and overfitting during model training. Furthermore, the data structure after PCA dimensionality reduction is more streamlined, making it more suitable for processing by the RF model. Therefore, this study demonstrates that the combination of PCA and RF with radiomic features not only enhances the prediction of invasiveness in lung adenocarcinoma but also provides an effective strategy for data processing and model fusion, especially in multimodal medical data analysis, with broad future applications. The model in this study provides an effective non-invasive tool for predicting the invasiveness of lung adenocarcinoma, aiding surgeons in formulating personalized surgical plans and treatment options for patients. This model offers a new approach for future early screening, particularly in the context of common clinical manifestations such as ground-glass nodules.

Despite employing multiple machine learning methods to predict the invasiveness of lung adenocarcinoma, this study has several limitations. First, the included patients were surgical candidates, leading to selection bias and a limited sample size. Moreover, due to objective constraints, the sample size of the external validation cohort is limited, which reduces the generalizability of the model. Thus, further large-scale, multicenter studies are required. Secondly, the study population was relatively homogeneous, consisting of early-stage lung adenocarcinoma patients; therefore, the results may not be generalizable to other populations, such as those with more advanced disease or different histological subtypes. Lastly, although we employed several machine learning methods, further model optimization and exploration of additional features remain critical areas for future research. Future research could consider expanding the sample size, incorporating a broader range of lung nodule imaging data, and utilizing more complex deep learning models to further enhance predictive accuracy. Additionally, the integration of multimodal imaging data (such as PET-CT, MRI or immune microenvironment standard markers) [55–57] would provide more information for predicting lung cancer invasiveness . Additionally, efforts can be made to explore the potential of radiomics-based approaches in the areas of radiotherapy, immunotherapy, and tumor microenvironment research for patients, which would further promote the development of personalized medicine.

## Supplementary Information

Supplementary Material 1.

Supplementary Material 2.

Supplementary Material 3.

Supplementary Material 4.

Supplementary Material 5.

Supplementary Material 6.

## Data Availability

The datasets generated and/or analyzed during the current study are not publicly available due to ongoing research using the same data. However, they are available from the corresponding author upon reasonable request, following approval from the institutional review board.

## Abbreviations

LA: Lung adenocarcinoma
GGN: Ground-glass nodules
CT: Computed tomography
ML: Machine learning
HR: CT High Resolution CT
PCA: Principal component analysis
RF: Random Forest
LR: Logistic Regression
AUC: Area Under the Curve
DCA: Decision Curve Analysis
SN: Solid nodules
PSN: Part-solid nodules
PGGN: Pure ground-glass nodules
MIA: Minimally invasive adenocarcinoma
LDCT: Low-dose spiral CT
ROI: Regions of interest
SHAP: SHapley Additive exPlanations
