# Supplementary material for: Machine learning-based prediction of invasiveness in lung adenocarcinoma presenting as ground-glass nodules using radiomics and clinical CT features
Source: BMC Cancer. 2025 Nov 3;25:1693. doi: 10.1186/s12885-025-14983-3 (PMC12581264; doi:10.1186/s12885-025-14983-3)
Supplement: Supplementary file 6 — Supplementary Material 6. [file 12885_2025_14983_MOESM6_ESM.docx]

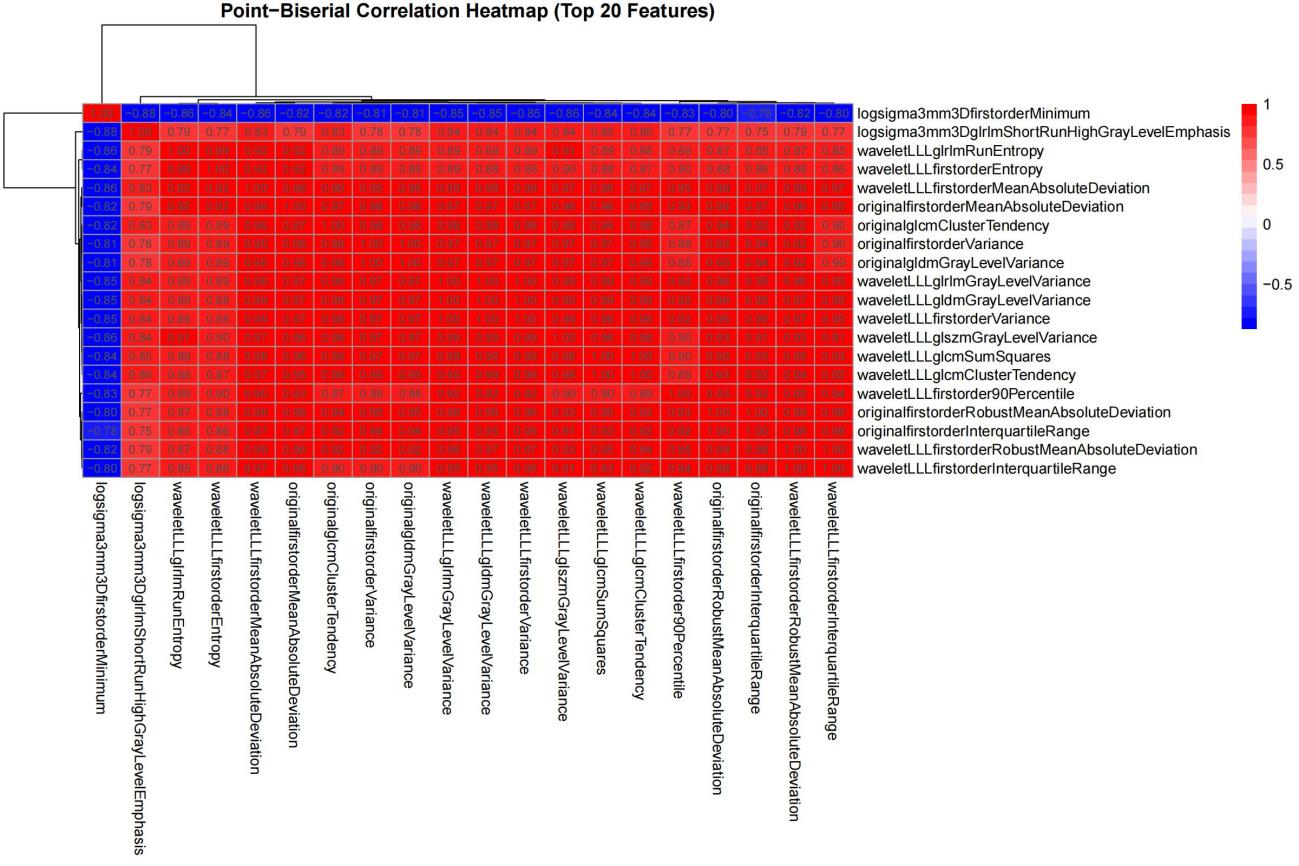


Figure S1. Heatmap of the top 20 most strongly correlated variables in primary cohort


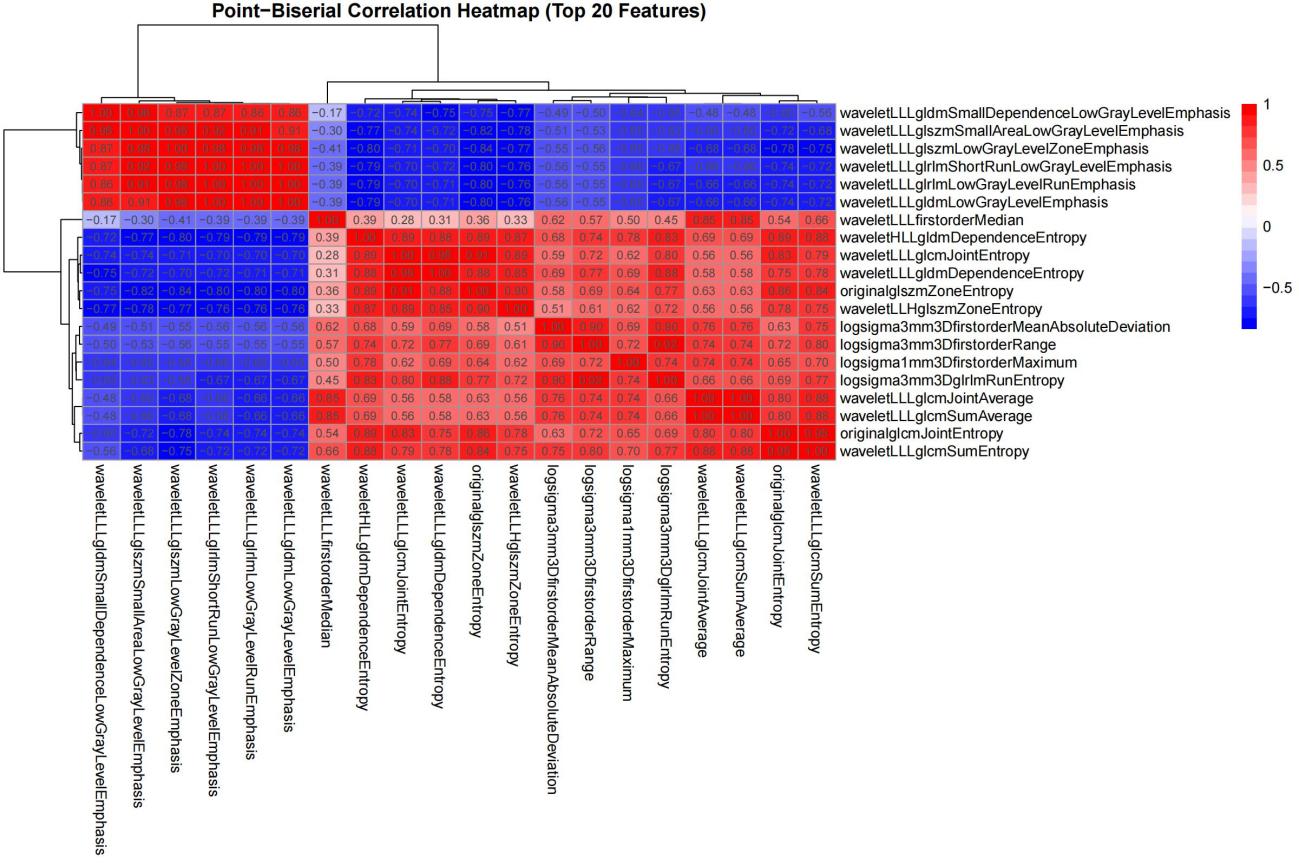


Figure S2. Heatmap of the top 20 most strongly correlated variables in validation cohort


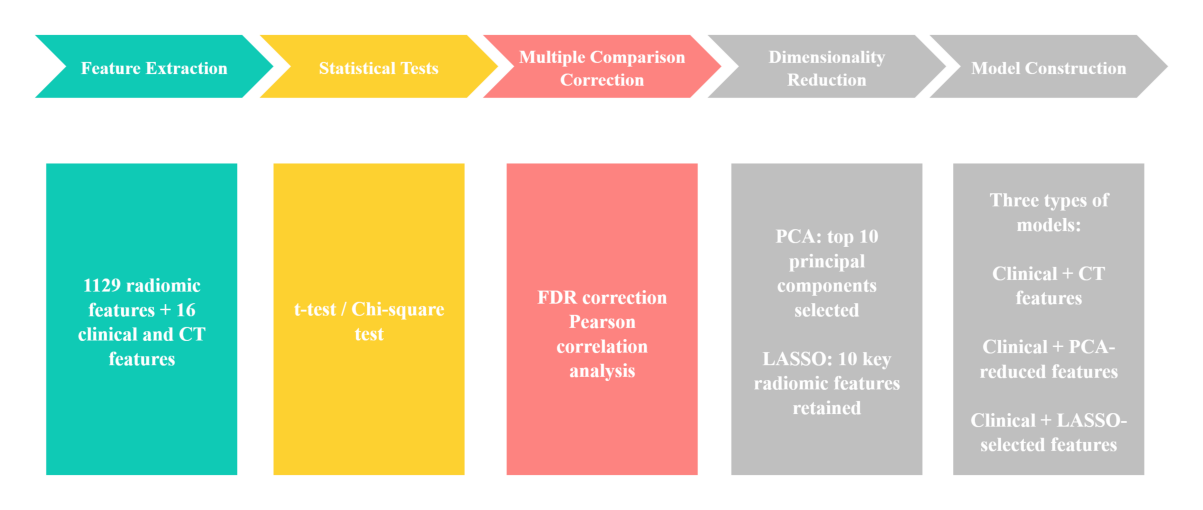


Figure S3. Feature selection flowchart


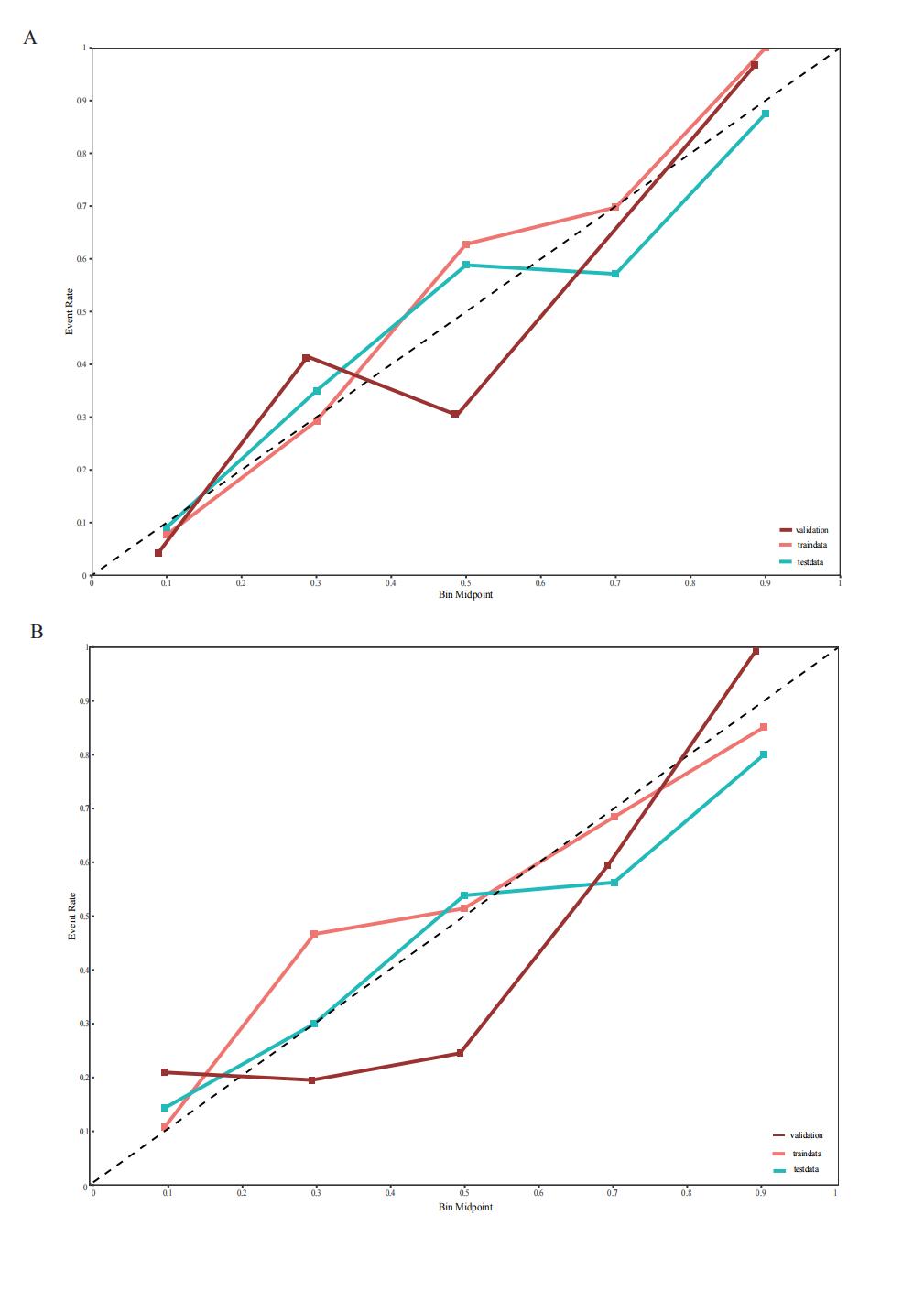


Figure S4. Calibration curve of the RF model

*A:Clinical CT Features-LASSO Radiomics Model,B:Clinical CT Features-PCA Radiomics Model,Light red (training cohort), blue (test cohort), dark red (external validation cohort)*


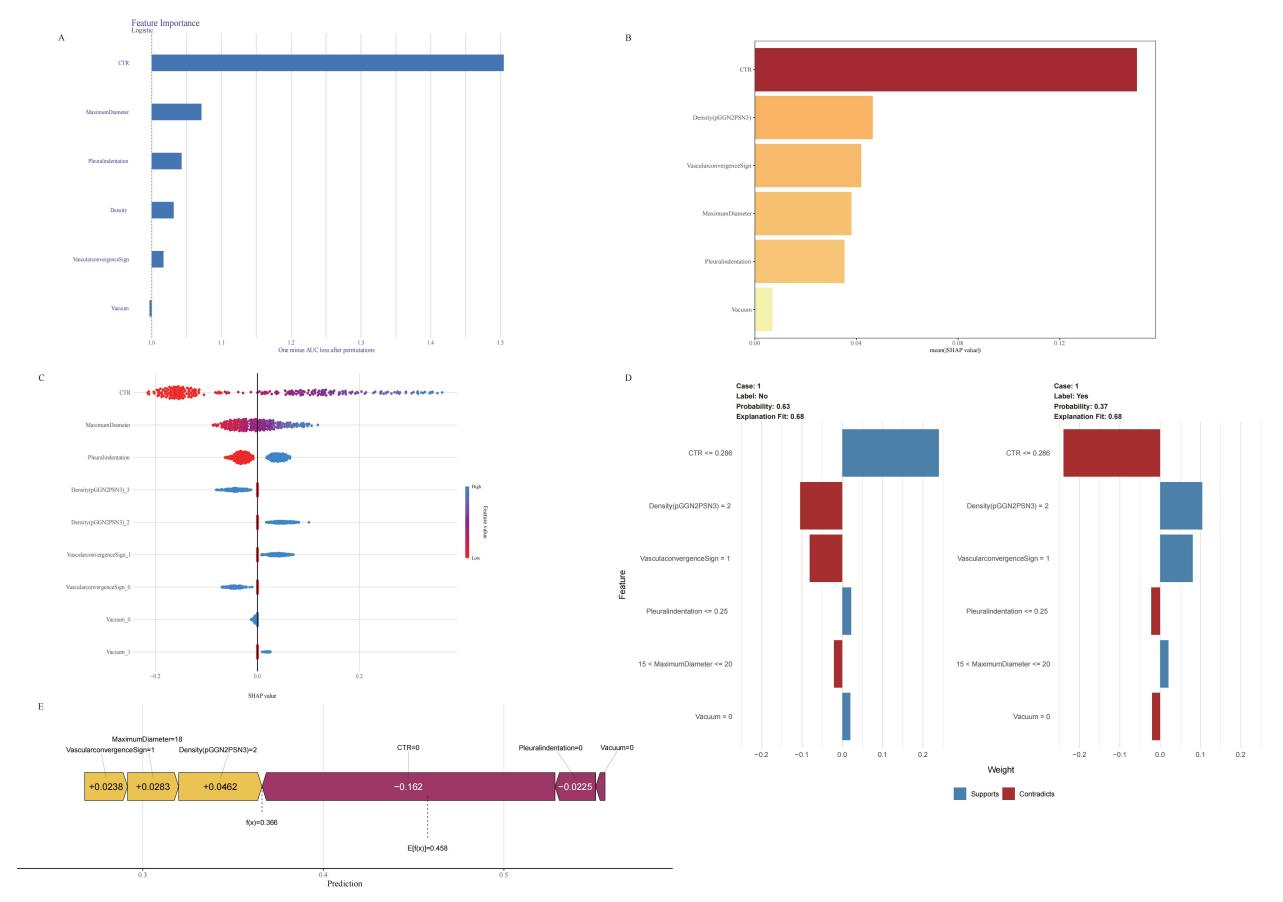


Figure S5 SHapley Additive exPlanations of Clinical CT Features

*Panel （LR）A: SHAP Feature Importance Plot.Panel B: SHAP Summary Plot.Panel C: SHAP Value Distribution Plot.Panel D: SHAP Decision Explanation Plot.Panel E: Local Interpretable Model-agnostic Explanations (LIME).0: Absence of Feature, 1: Presence of Feature.*
